# Gallstone Ileus: A Rare Case of Intestinal Obstruction

**DOI:** 10.7759/cureus.99777

**Published:** 2025-12-21

**Authors:** Joana Rodrigues Ferreira, Tomás Matos Nunes, Madalena Costa, Rafael Carvalho, José Guilherme Cardoso

**Affiliations:** 1 General Surgery, Unidade Local de Saúde de Lisboa Ocidental, Lisbon, PRT; 2 Bariatric Surgery, Centro Hospitalar de Lisboa Ocidental, Lisbon, PRT

**Keywords:** cholecysto-duodenal fistula, enterolithotomy, gallstone ileus, small bowel obstruction, surgical case reports

## Abstract

Gallstone ileus is a rare mechanical bowel obstruction caused by the impaction of a gallstone in the GI tract, most frequently in the terminal ileum and the ileocecal valve, secondary to a cholecystoenteric fistula. It predominantly affects elderly women with multiple comorbidities, which, together with non-specific and intermittent symptoms, delay the diagnosis. Imaging studies play a crucial role in diagnosis. Despite that, gallstone ileus continues to be associated with relatively high rates of morbidity and mortality. The optimal management of acute gallstone ileus remains controversial. The selection of a surgical procedure is primarily influenced by the clinical condition. Currently, enterolithotomy remains the most common and safest surgical method, with spontaneous closure of the fistulous tract. One-stage procedure is time-consuming, technically demanding, and is independently associated with a higher prevalence of mortality. Delayed cholecystectomy should be reserved for patients with persistent symptoms. This report describes the case of an 84-year-old man, partially dependent, who presented to the emergency department with a four-day history of fecaloid vomiting, food intolerance, and epigastric pain. The prompt evaluation and the high index of suspicion allowed for the guidance of imaging examination, correct diagnosis, and the surveillance and optimization in an intensive care unit. Despite the conservative therapy chosen, the patient has an unfavorable progression, indicating mechanical intestinal occlusion due to biliary ileus with possible ischemic involvement. A longitudinal enterolithotomy was performed. During hospitalization, the patient was subjected to an upper GI endoscopy with electrohydraulic lithotripsy that allowed the fragmentation of the stone. The patient progressed favorably, without any new episodes of biliary colic or intestinal obstruction within six months postoperatively. This case represents the importance of opting for less invasive approaches in frail patients with good long-term results.

## Introduction

Gallstone ileus refers to a mechanical obstruction of the bowel resulting from the entrapment of a gallstone in the GI tract, secondary to a cholecystoenteric fistula [1,2]. It is an uncommon yet significant complication of cholelithiasis, representing 1-4% of all causes of intestinal obstruction [1,3]. Gallstone ileus is more common in elderly women with multiple comorbidities, which, together with non-specific and intermittent symptoms, delays the diagnosis [3,4]. As a consequence, gallstone ileus is still correlated with relatively high levels of morbidity and mortality [3,4]. The optimal management of gallstone ileus remains controversial [5]. We report a rare case of gallstone ileus, without symptom recurrence after a successful surgical treatment with enterolithotomy alone.

## Case presentation

An 84-year-old male patient, partially dependent, with hypertension, previously submitted to bilateral inguinal hernioplasty and radical prostatectomy, was brought to the ER due to a four-day history of vomiting with fecaloid content, food intolerance, and epigastric abdominal pain, with no changes in his bowel habits.

On physical examination, he demonstrated hemodynamic stability, exhibiting signs of dehydration. The abdomen was distended, presenting diffuse pain upon deep palpation, without signs of peritoneal irritation on percussion. Digital rectal examination was unremarkable. Laboratory tests revealed leukocytosis, acute kidney injury of probable prerenal etiology, electrolyte disturbances (hyponatremia and hyperkalemia), and elevated C-reactive protein (Table 1).

**Table 1:** Temporal evolution of laboratory characteristics during hospitalization Laboratory findings show worsening signs of dehydration (acute kidney injury and electrolyte disturbances) and inflammatory parameters (leukocytosis and elevated C-reactive protein)

Parameters	Patient values at admission	Patient values in ICU	Reference range
Red blood cell count	5.01	4.97	4.3-5.9 x 10^12^/L
Hemoglobin	13.6	14.1	13.5-17.5 g/dL
Hematocrit	0.413	0.456	0.406-0.504
Mean corpuscular volume	82.4	80.7	80-96 fL
Mean corpuscular hemoglobin	27.1	26.7	27.3-33.7 pg
Mean corpuscular hemoglobin concentration	329	320	328-354 g/dL
Red cell distribution width	15.4	15	11.5-14.5%
Platelet count	148	145	150-400x 10^9^/L
Complete white blood cells	12.9	16.1	4-10x10^9^/L
Neutrophils	70.4	71	40-80%
Lymphocytes	17.9	18.2	20-40%
Monocyte	11.5	11	2-11.7%
Eosinophils	0.1	1	1-6%
Basophils	0.1	0.2	0-2%
Urea	204	196	17-49 mg/dL
Creatinine	5.53	6.68	0.7-1.2 mg/dL
Albumin	3.2	2.9	3.5-5.2 g/dL
Bilirubin total	0.4	0.42	<1.4 mg/dL
Sodium	132	130	136-145 mmol/L
Potassium	4.8	5.38	3.5-5.1 mmol/L
Chloride	90	101	98-107 mmol/L
Calcium	7.8	7.9	8.8-10.2 mg/dL
Phosphorus	5.4	4.4	2.5-4.5 mg/dL
Magnesium	2.5	2.1	1.6-2.4 mg/dL
C-reactive protein	21.5	29.7	<0.5 mg/dL

Abdominal X-ray showed air-fluid levels. He underwent a non-contrast computed tomography (CT) of the abdomen that showed a diffuse dilation of the small bowel with a non-obstructive hyperdense image in the lumen of the third duodenal portion. A 5-mm cholecystoenteric fistula between the gallbladder fundus and the second portion of the duodenum was identified. Additionally, it was associated with extensive pneumobilia and a transition point in the mesentery with an abrupt reduction of bowel caliber.

**Figure 1:**
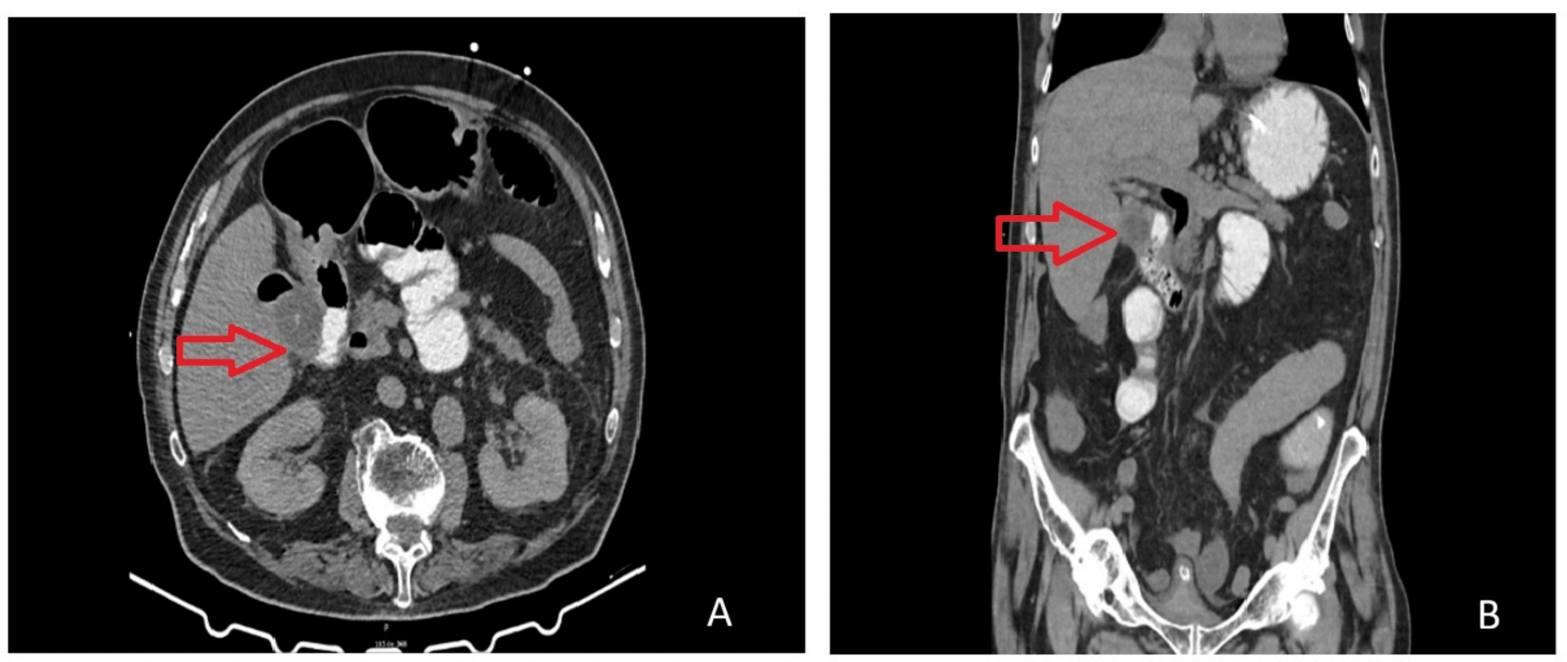
Axial and coronal section of simple abdominal CT Repeat CT shows diffuse dilation of the small bowel  with a cholecystoenteric fistula between the gallbladder fundus and the second duodenal portion (red arrow)

The patient underwent gastrografin, with apparent progression of the contrast agent without evident stoppage. Initially, conservative therapy was chosen, and empirical antibiotic therapy was initiated. He remained under surveillance in the ICU, presenting continuous biliary drainage of approximately 1000 mL/24 hours and progressive worsening of inflammatory markers on Day 2 (Table 1). A repeat abdominal CT scan with oral contrast revealed the absence of contrast progression, consistent with mechanical small bowel obstruction due to gallstone ileus and possible ischemic involvement (Figure 1).

**Figure 2:**
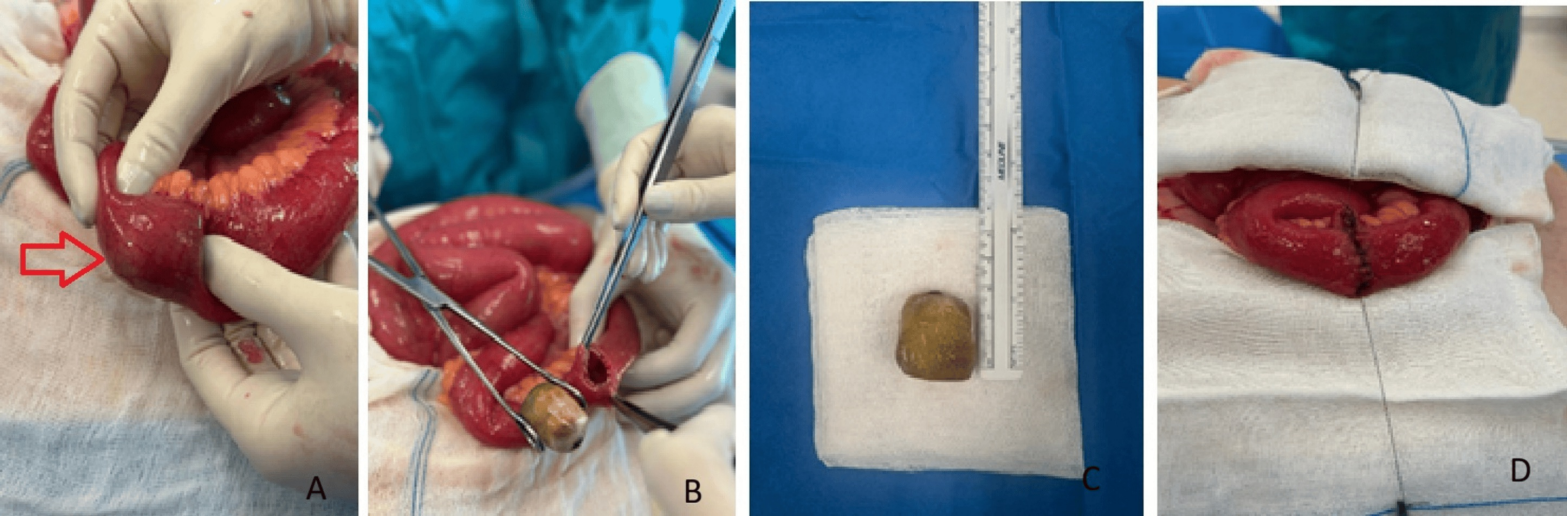
Intraoperative findings (A) Dilated bowel loop with stone inside (red arrow), with no signs of ischemia. (B) Longitudinal enterotomy with stone extraction. (C) 2.9 x 2.6 cm stone. (D) Enterorrhaphy with absorbable suture in two planes

During the exploratory laparotomy, dilated intestinal loops were found 100 cm from the ileocecal valve, without signs of ischemia. An inflammatory mass was visible in the region of the hepatic and gallbladder hilum, so, given the clinical condition, it was decided not to approach the area. A longitudinal enterotomy in the antimesenteric border was performed, leading to the extraction of an approximately 2.5 cm stone that occluded the intestinal lumen entirely, without vascular compromise (Figures 2A-2C). Subsequently, a two-layer closure with absorbable sutures was performed (Figure 2D). Further investigation of the intestine and colon was conducted, yet no further stones were identified.

**Figure 3:**
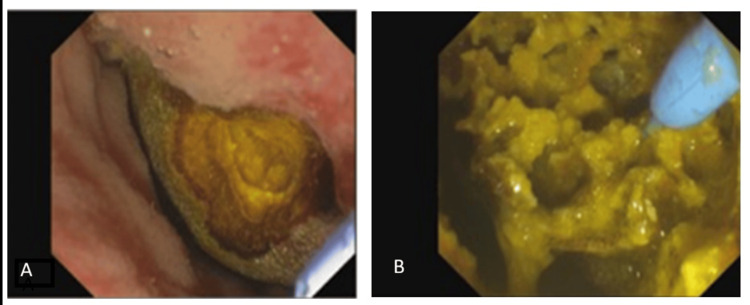
Upper gastrointestinal endoscopy and electrohydraulic lithotherapy Upper gastrointestinal endoscopy that revealed the cholecystoduodenal fistula with a large stone protruding into the duodenal lumen (A), and electrohydraulic lithotherapy (B) was performed which allowed the fragmentation of the stone

During hospitalization, an upper gastrointestinal endoscopy was performed, revealing a cholecystoduodenal fistula in the proximal second portion of the duodenum, with a large stone protruding into the duodenal lumen and reducing its size (Figure 3). Electrohydraulic lithotripsy was tried, which allowed the fragmentation of the 3 cm stone (two-thirds of the total volume by visual estimation).

The patient progressed favorably, tolerated diet progression while maintaining intestinal transit, and was discharged home on the seventh postoperative day. At the medical consultation, he tolerated a low-fat diet, without any new episodes of biliary colic or intestinal obstruction. Six months postoperatively, he underwent a reassessment CT abdominal scan that highlighted persistent parietal hyperemia of the bile ducts associated with pneumobilia, suggesting a persistent fistula, without associated gallstones or changes in caliber or uptake in the small intestine or colon.

## Discussion

Gallstone ileus is a rare but serious complication of cholelithiasis, often preceded by an episode of cholecystitis, in which the inflammation leads to the formation of a fistula to the adjacent GI tract. Gallstone ileus can also arise as a complication of an endoscopic retrograde cholangiopancreatography or an endoscopic sphincterotomy [6], and the consequence of loose stones in the abdominal cavity after a laparoscopic cholecystectomy [5]. The gallstone must be superior to 2-2.5 cm in diameter to cause obstruction, typically found in the terminal ileum and the ileocecal valve as a result of their constricted lumen and potentially diminished peristalsis activity (60.5% of cases) [7,8].

Presentation is typically non-specific, with vague, intermittent symptoms, generally associated with an advanced age and several comorbidities, and may be the result of a delay in diagnosis [8]. Without early treatment, the patient will progress with ischemia and subsequent perforation and peritonitis, with a mortality of 12-27% [5]. Imaging studies have a crucial role in diagnosis, especially with the widespread use of CT. Plain radiographic findings, such as pneumobilia, dilated loops, and ectopic stone (Rigler’s triad) are only observed in 15% of cases [9,10].

There is no consensus on the best approach and surgical procedure, and the management of gallstone ileus remains controversial. There are several approaches described in the literature: (i) conservative treatment with nasogastric decompression; (ii) enterolithotomy or bowel resection alone; (iii) one-stage surgery: enterolithotomy, cholecystectomy, and fistula closure; (iv) two-stage surgery: delayed cholecystectomy and fistula closure four to six weeks after enterolithotomy [7,11,12]. In over 50% of cases, the fistulous tract undergoes spontaneous closure [11].

The determination of the surgical procedure is predominantly based on the clinical status, usually prioritizing techniques that are less invasive. Currently, enterolithotomy remains the most common and safest surgical method, with a low incidence of complications and low morbidity and mortality [11,13]. In the presence of residual stones, 5% of patients go on to develop biliary symptoms within six months of the index presentation, and there is a potential risk of patent fistula reflux resulting in biliary malignancy [8,11,14]. Laparoscopy-assisted methods have been described; however, they are technically more demanding [15]. Additionally, there are cases reported in the literature of endoscopic extraction, like in Bouveret’s syndrome [16]. One-stage procedure is time-consuming, technically demanding, and was independently associated with a higher prevalence of mortality [17]. Delayed cholecystectomy should be reserved only in cases of symptom persistence. 

## Conclusions

Gallstone ileus is a rare entity that exhibits a gradual onset with unclear signs and symptoms. For that reason, accurate diagnosis is crucial, and a significant clinical suspicion is needed when there is a documented history of acute or recurring cholecystitis. Taking into account the advanced age and comorbidities of most patients, relief of obstruction by enterolithotomy alone in the emergency setting is recommended. One-stage procedure has a number taking more time, being more technically challenging, and being associated with a higher prevalence of mortality. Delayed cholecystectomy should be reserved only in cases of symptom persistence. This case highlights the importance of opting for less invasive approaches in frail patients with good long-term results.
